# 3D assessment of alveolar bone alterations in orthodontic movement among Indians

**DOI:** 10.6026/97320630019764

**Published:** 2023-06-30

**Authors:** Sweta Gupta, Vinit Kumar Singh, Shreya Pandey, Sandeep Singh, Avinash Jaiswal, Chinmaya Hawaldar

**Affiliations:** 1 Department of Orthodontics and Dentofacial Orthopaedics, Patna Dental College and Hospital, Patna, Bihar, India; 2 Department of Orthodontic and Dentofacial Orthopedic, Vananchal Dental College, Garhwa, Jharkhand, India; 3 Department of Orthodontic and Dentofacial Orthopedic, Dental College Azamgarh, Azamgarh, UP, India; 4 Department of Orthodontic and Dentofacial Orthopedic, Terna Dental College and Hospital, Navi Mumbai, Maharashtra, India

**Keywords:** 3D, alterations of alveolar bone, apical root resorption, clear aligners, and fixed appliances

## Abstract

Apical root resorption, which is characterised as a biological or abnormal phenomenon that shortens the length of the root apex, is additional typical iatrogenic impact of orthodontic tooth movement that may jeopardise the effectiveness of treatment and tooth lifespan. The main goals of the current retrospective investigation were to assess the dimensions of alveolar bone alterations that come along with orthodontic movement and to look into the frequency and extent of resorption of root in maxillary incisors across categories that were similarly managed with clear aligners (OCA) and fixed appliances (OFA) using CBCT. The study included 50 subjects who were divided into two categories with 25 study subjects in each category. Category OFA: Subjects receiving OFA (n=25). A CBCT scan was used to get three-dimensional pictures at the beginning of therapy as well as at the end of therapy. The overall resorption of root at apical region in OFA group was 0.63±0.21 mm. The overall resorption of root at apical region in OCA group was 0.32 ±0.36 mm. The difference in observation was statistically significant (p= 0.000) with reduced resorption of root at apical region in clear aligners. It was concluded that the decrease in thickness of alveolar bone was greater in orthodontic fixed appliances group as compared to clear aligners. The resorption of root at apical region was lesser in clear aligners group as compared to fixed appliances.

## Background:

Fixed orthodontic equipment (FA) therapy became the most widely used orthodontic device as a result of its effectiveness [1]. The appliance's look and the patient's capacity to maintain good dental health, however, frequently prevent patients from accepting the appliance [2, 3, 4]. The dentistry industry is rapidly changing, hastening the transition to patient-focused techniques and necessitating the introduction of novel appliances for orthodontics that satisfy the needs of both patients and treating physicians. Recently, clear aligner (OCA) therapy was used in dentistry as a more aesthetically pleasing and cosy substitute for fixed appliance (OFA). The "ClinCheck" 3D dental planning programme from Align Technology allows dentists to virtually plan therapy and examine every shift of the teeth until final outcome. A wide variety of orthodontic problems, have recently been treated with CA [5]. However, the research continues to debate its precision in clinical practice and conformity with the specified actions on the programme [6]. Despite the drawbacks of both devices, earlier research has demonstrated that both could be utilised to address situations of light and intermediate overcrowding [7]. Apical root resorption (ARR), which is characterised as a biological or abnormal phenomenon that shortens the length of the root apex, is additional typical iatrogenic impact of orthodontic tooth movement (OTM) that may jeopardise the effectiveness of treatment and tooth lifespan [8,9]. Almost often, orthodontically triggered reactive resorption of root (OIIRR) takes place, and the severity of it fluctuates from tooth to tooth [10]. In a recently released comprehensive review, it was discovered that FA treatment causes the OIIRR in the upper incisors to rise. The upper jaw lateral incisors in the OCA method had lesser resorption of the roots than the FA system, according to a recently published comprehensive review [11], while other investigations discovered that CA has a reduced incidence and extent of resorption of roots than OFA [12]. Depending on the type of detecting technique utilised, diagnostic precision varies [13].Prior research suggested that CBCT, which aids in quantitatively evaluation of alveolar bone and the length of roots with outstanding precision and accuracy while also being extremely repeatable [14] and demonstrating outstanding sensitivity as well as precision [15], has inherent advantages over traditional two-dimensional (2D) imaging due to enlargement and deformation of images. To guarantee the protection of patients, extra care needs to be undertaken when utilising CBCT, such as utilising a CBCT with restricted field of view as well as wearing protective gear [16,17,18, 19,20,21,22]. As a result, the main goals of the current retrospective investigation were to assess the dimensions alveolar bone alterations that come along with orthodontic movement (OTM) and to look into the frequency and extent of OIIR in maxillary incisors across categories that were similarly managed with OCA and OFA. The secondary goal of the current research was to examine the resorption of root and post-operative dimensional alterations of alveolar bone alterations in maxillary incisors using either approach through CBCT.

##  Methods and Materials:

## Calculation of sample size:

In accordance with the investigation by Li Yu *et al*.[23], who found maxillary resorption of root in upper canine as of 1.53±1.92 mm and 0.14±0.53 mm in the OFA and OCA categories, respectively, the number of participants was calculated using G*power programme (version 3.0.10) with 0.05 alpha value and 85% power. Power analysis indicated a sample size of at least 23 participants. For each research category, the participant population was raised to 25 patients. The study included 50 subjects who underwent orthodontic treatment either with OFA or OCA. They were divided into two categories with 25 study subjects in each category.

Category OFA: Subjects receiving OFA (n=25)

Category OCA: Subjects receiving OCA (n-25)

Records of patients undergoing orthodontic treatment were examined between March 2, 2019, and May 2, 2023. One skilled operator used either traditional OFA or OCA to treat patients.

## The following were the inclusion criteria:

[1] Patients above the age of 18,

[2] Light to medium crowdedness,

[3] Non-extraction therapy,

[4] Complete permanent dentition, but without third molars, and

[5] Outstanding pre-treatment (D0) documents and post-treatment (D1) documents, including CBCT scans, pictures, and modeling casts, that were acquired during the course of their orthodontic evaluation and therapeutic strategy.

##  Exclusion standards comprised:

[1] Patients having a history of trauma or recent maxillary incisor root canal therapy,

[2] Prior early intervention or thorough orthodontic care,

[3] Past maxillofacial injuries,

[4] Birth defects, including cleft lip and/or palate, maxillary hypoplasia,

[5] Systemic illnesses,

[6] Smokers,

[7] Loss of adhesion to the periodontium,

[8] Indications of inflammatory root resorption in the past.

Any disclosed active inflammatory disorders showing insufficient favorable periodontal structures were eliminated depending on the thorough periodontal assessment performed prior to orthodontic therapy that was thoroughly documented in the periodontal assessment section of the patients' documents. The present investigation comprised just patients with healthy periodontium prior to therapy. The case complexity in every category was assessed using the American Board of Orthodontics (ABO) disparity scale (DI) [21]. In order to maximise the comparison of the two investigated categories, the level of complexity of the cases in every category was measured using the ABO discrepancy index, and cases with substantial scores were eliminated.50 patients overall who met the eligibility requirements were split into two equally matched categories (OCA and OFA). 25 patients, who received Invisalign® treatment through Align Technology USA, were a part of the CA subgroup. The fixed orthodontic appliance used in the OFA group on 25 patients was by 3 M Unitek®, California, and USA.A CBCT scan was used to get three-dimensional pictures at the beginning of therapy as well as at the end of therapy. The preoperative imaging settings of CBCT were comparable to the post-treatment imaging settings. These were FOV of 230 x 170 mm, kvp was120 kV, current was 5 mA, voxel size of 3mm.The patient's teeth were almost completely inter cusped and patients were seated in upright position. The midsagittal axis was kept at 90 degree with the floor, and the Frankfort horizontal plane remained parallel to the ground. In order to do the three dimensional analysis, the acquired CBCT scan results were converted into the DICOM data file format and entered into the Bluesky software programme (USA).The axial section was used to measure the thickness of alveolar bone at the labial surface of teeth and palatal surface of teeth. Measurements were carried at at three distinct levels at intervals of 3 mm from CEJ (L1), 6 mm from CEJ (L2), and 9 mm from the CEJ (L3) at maxillary incisors. The distance between crest of alveolar ridge and CEJ on labial surface, and palatal surface was considered as the height of bone. To gauge length of root preoperatively and post operatively, the length between the created CEJ line and the most apical location of root was calculated. The angle between the maxillary incisor (MI) and the palatal axis (MI-PA) was used to assess the inclination. On every CBCT images taken before beginning of therapy, height of alveolar bone, length of root, thicknesses of alveolar bone, and inclinations of the incisors of maxilla were all quantified. Without gaining access to the preoperative measures, post-treatment evaluations were carried out two weeks afterwards. Specialists of Oral and maxillofacial radiology have supervised and guided all measurements throughout. The degree of severity of resorption of roots was graded according to Sharpe's technique [26]. To guarantee that the investigator was unaware of the treatment categories during data collection and evaluation, study models as well as information were encrypted.

## Statistical analysis:

Each outcome was examined independently. Using SPSS, edition 19.0, descriptive statistics which refers to means as well as standard deviations were determined for every category. Initially, one-way ANOVA was conducted applying Tukey's HSD for post-hoc analyses. In order to test for disparities in variances, an independent-samples t-test was performed for each result. Numerous comparisons were taken into account by applying the Bonferroni adjustment. It should be observed that the outcomes of the one-way ANOVA statistical test and Tukey's HSD statistical test consistently resembled the outcomes of the independent-samples t-test statistical test.

The mean value of pretreatment bone thickness at labial surface at L1 in OFA group was 0.88±0.38 mm. The mean value of post treatment bone thickness at labial surface at L1 in OFA group was 0.71±0.59 mm. The difference in findings was statistically significant (p=0.001). The mean value of pretreatment bone thickness at labial surface at L2 in OFA group was 1.11±0.30mm. The mean value of post treatment bone thickness at labial surface at L2 in OFA group was 1.10±0.69 mm. The mean value of pretreatment bone thickness at labial surface at L3 in OFA group was 1.08±0.75 mm. The mean value of post treatment bone thickness at labial surface at L3 in OFA group was1.28±1.11 mm. overall pretreatment average value of bone thickness at labial side of maxillary incisors was 1.08±0.61mm while post treatment average value of bone thickness at labial side of maxillary incisors was 1.10±0.85 mm.

The mean value of pretreatment bone thickness at palatal surface at L1 in OFA group was 1.66±0.79 mm. The mean value of post treatment bone thickness at palatal surface at L1 in OFA group was 1.22±0.85 mm. The difference in findings was statistically significant (p=0.001). The mean value of pretreatment bone thickness at palatal surface at L2 in OFA group was 3.26±1.08mm. The mean value of post treatment bone thickness at palatal surface at L2 in OFA group was 2.50±1.25 mm. The difference in findings were statistically significant (p=0.000). The mean value of pretreatment bone thickness at palatal surface at L3 in OFA group was 4.84 ±1.70 mm. The mean value of post treatment bone thickness at palatal surface at L3 in OFA group was 4.10±2.17 mm. The difference in finding was statistically significant (p=0.12).Overall pretreatment average value of bone thickness at palatal side of maxillary incisors was 3.34±1.85 mm while post treatment average value of bone thickness at labial side of maxillary incisors was 2.87±1.98 mm. The difference in finding was statistically significant. (p= 0.000) (Table 1).

The mean value of pre-treatment bone thickness at labial surface at L1 in OCA group was 1.52±0.63 mm. The mean value of post treatment bone thickness at labial surface at L1 in OCA group was 0.71±0.66 mm. The mean value of pre-treatment bone thickness at labial surface at L2 in OCA group was 0.90±0.48 mm.. The mean value of post treatment bone thickness at labial surface at L2 in OCA group was 0.96±0.55 mm. The mean value of pre-treatment bone thickness at labial surface at L3 in OCA group was 1.11±0.42 mm.. The mean value of post treatment bone thickness at labial surface at L3 in OCA group was 0.92±0.74 mm. The difference in findings was statistically significant. (p= 0.040). Overall pre-treatment average value of bone thickness at labial side of maxillary incisors was 0.95±0.46 mm while post treatment average value of bone thickness at labial side of maxillary incisors was 0.86±0.66 mm. The mean value of pre-treatment bone thickness at palatal surface at L1 in OCA group was 1.66±0.79 mm. The mean value of post treatment bone thickness at palatal surface at L1 in OCA group was 1.22 ±0.81 mm. The difference in findings was statistically significant (p=0.001). The mean value of pre-treatment bone thickness at palatal surface at L2 in OCA group was 3.01±1.25mm. The mean value of post treatment bone thickness at palatal surface at L2 in OCA group was3.06±1.24 mm. The mean value of pre-treatment bone thickness at palatal surface at L3 in OCA group was 4.75±1.97 mm. The mean value of post treatment bone thickness at palatal surface at L3 in OCA group was 4.82±1.69 mm. Overall pre-treatment average value of bone thickness at palatal side of maxillary incisors was 3.16±1.93 mm while post treatment average value of bone thickness at labial side of maxillary incisors was 3.04±1.99 mm (Table 2).

The mean value of pre-treatment height of bone at labial surface in OFA group was 2.03±0.60 mm. The mean value of post-treatment height of bone at labial surface in OFA group was 2.97 ±1.61 mm. The difference in findings was significant statistically. (p= 0.000). The mean value of pre-treatment height of bone at palatal surface in OFA group was 1.52 ±0.77 mm. The mean value of post-treatment height of bone at palatal surface in OFA group was 2.41 ±1.89 mm. The difference in findings was significant statistically. (p= 0.000).The mean value of pre-treatment height of bone at labial surface in OCA group was 2.11 ±0.66 mm. The mean value of post treatment height of bone at labial surface in OCA group was 3.21±2.46 mm. The difference in findings was significant statistically. (p= 0.000). The mean value of pre-treatment height of bone at palatal surface in OCA group was 2.21±0.66 mm. The mean value of post treatment height of bone at palatal surface in OCA group was 3.11 ±2.46 mm. The difference in findings was significant statistically. (p= 0.000). (Table 3)

The mean value of pre-treatment resorption of root at apex in OFA was 11.83±1.79 mm. The mean value of post-treatment resorption of root at apex in OFA was 11.25±2.23 mm. The difference in findings was significant statistically. (p= 0.000). The mean value of pre-treatment inclination (MI-PA) in OFA was 119.35 ± 6.76°. The mean value of post-treatment inclination (MI-PA) in OFA was 119.58 ± 8.49°.The mean value of pre-treatment resorption of root at apex in OCA was 12.53±1.89 mm. The mean value of post-treatment resorption of root at apex in OCA was 11.21±1.71 mm. The difference in findings was significant statistically. (p= 0.000). The mean value of pre-treatment inclination (MI-PA) in OCA was 118.74 ±7.0 °. The mean value of post-treatment inclination (MI-PA) in OCA was 114.27 ± 5.53°. The difference in findings was statistically significant. (p= 0.044) (Table 4).

The difference in overall pre-treatment bone thickness and post treatment bone thickness in OFA group was 0.21 ± 0.93 mm. The difference in overall pre-treatment bone thickness and post treatment bone thickness in OCA group was -0.01±0.47 mm. The difference in observation was significant statistically. (p=0.000). There was decrease in thickness of bone in fixed appliance group after therapy while in contrast there was increase in thickness of alveolar bone in clear aligner group after therapy. The difference in overall pre-treatment height of alveolar bone and post treatment height of alveolar bone in OFA group was 0.93±1.47 mm. The difference in overall pre-treatment height of bone and post treatment height of bone in OCA group was 0.91±2.44 mm. The difference in observation was not significant statistically (p=0.160).The overall resorption of root at apical region in OFA group was 0.63±0.21 mm. The overall resorption of root at apical region in OCA group was 0.32 ±0.36 mm. The difference in observation was statistically significant (p= 0.000) with reduced resorption of root at apical region in clear aligners. The overall change in inclination in OFA group was -1.24 ± 8.11°. The overall change in inclination in OCA group was 4.63 ±8.14°. The difference in observation was statistically significant (p= 0.000) (Table 5).

## Discussion:

A common iatrogenic effect of OTM that may compromise the success of therapy and tooth longevity is apical root resorption (ARR), which is defined as a biological or pathological occurrence that reduces the length of the root apex [15,16]. The frequency and severity of orthodontically induced irreversible root resorption (OIIRR) vary from tooth to tooth [17]. The primary objectives of the current retrospective analysis were to evaluate the dimensions of alveolar bone modifications associated with OTM and to investigate the prevalence and severity of OIIR in categories of maxillary incisors that were similarly handled with OCA and OFA. In order to assess the resorption of root and post-operative dimensional changes of alveolar bone in maxillary incisors utilising either technique by CBCT, this study's secondary objective was established. In this study the prevalence of resorption of root at apical region in OFA was 82% while it was 68% in OCA. More severe resorption of root at apical region was observed in OFA as compared to OCA. The difference in overall pre-treatment bone thickness and post treatment bone thickness in OFA group was 0.21 ± 0.93 mm. The difference in overall pre-treatment bone thickness and post treatment bone thickness in OCA group was -0.01±0.47 mm. The difference in observation was significant statistically. (p=0.000). There was decrease in thickness of bone in fixed appliance group after therapy while in contrast there was increase in thickness of alveolar bone in clear aligner group after therapy. The OIIRR in the upper incisors increases after FA treatment, according to a comprehensive evaluation that was just published [21]. According to a recently published comprehensive review conducted by Gandhi *et al*. [22], the upper jaw lateral incisors in the OCA method had less root resorption than the FA system, while other investigations found that CA has a lower incidence and extent of root resorption than OFA [23, 24, 25].In this study, the difference in overall pre-treatment height of alveolar bone and post treatment height of alveolar bone in OFA group was 0.93±1.47 mm. The difference in overall pre-treatment height of bone and post treatment height of bone in OCA group was 0.91±2.44 mm. The difference in observation was not significant statistically (p=0.160).Diagnostic accuracy varies depending on the type of detecting technique used [18].Prior studies suggested that CBCT has inherent advantages over conventional two-dimensional (2D) imaging due to enlargement and deformation of images. CBCT aids in quantitatively evaluating alveolar bone and the length of roots with outstanding precision and accuracy while also being extremely repeatable [19] and demonstrating outstanding sensitivity as well as precision [20]. When using CBCT, further precautions must be taken to ensure the safety of the patients, such as donning protective gear and using a CBCT with a limited field of vision. Due to its efficiency, fixed orthodontic equipment (FA) therapy rose to become the most used orthodontic device [21]. However, patients frequently refuse to accept the appliance because of how it looks and their ability to maintain good dental health [22]. The practice of dentistry is fast evolving, hastening the shift to patient-centred approaches and calling for the development of novel orthodontic appliances that meet the demands of both patients and treating physicians. Clear aligner therapy (CA) has recently become popular in dentistry as a more aesthetically acceptable and comfortable alternative to fixed appliances (FA). With Align Technology's "ClinCheck" 3D dental planning programme, practitioners may digitally plan treatment and monitor each tooth shift up until the final result. In this study the overall resorption of root at apical region in OFA group was 0.63±0.21 mm. The overall resorption of root at apical region in OCA group was 0.32 ±0.36 mm. The difference in observation was statistically significant (p= 0.000) with reduced resorption of root at apical region in clear aligners. The overall change in inclination in OFA group was -1.24 ± 8.11°. The overall change in inclination in OCA group was 4.63 ±8.14°. The difference in observation was statistically significant (p= 0.000). (Table 5) The CBCT imaging parameters used during preoperative and post-treatment phases were comparable. The FOV was 230 x 170 mm, the kvp was 120 kV, the current was 5 mA, and the voxel size was 3 mm. The patient was sat in an upright position with virtually fully inter cusped teeth. The Frankfort horizontal plane remained parallel to the ground, and the midsagittal axis was maintained at a 90-degree angle with the floor. The collected CBCT scan results were converted into DICOM data files and entered into the Bluesky software programme (USA) in order to do the three-dimensional analysis.The thickness of alveolar bone at the labial surface and palatal surface of teeth was measured using the axial section. At the maxillary incisors, measurements were made at three different levels at intervals of 3 mm from the CEJ (L1), 6 mm from the CEJ (L2), and 9 mm from the CEJ (L3). The height of the bone was calculated as the distance between the crest of the alveolar ridge and the CEJ on the labial surface and palatal surface. The distance between the newly constructed CEJ line and the root's most apical point was measured in order to determine the root's length both before and after surgery. The inclination was measured using the angle between the maxillary incisor (MI) and the palatal axis (MI-PA).Height of alveolar bone, length of root, thicknesses of alveolar bone, and inclinations of the maxillary incisors were all measured on each CBCT image taken prior to the start of therapy. Two weeks after the procedure, post-treatment evaluations were conducted without having access to the preoperative measures. All measurements have been inspected and directed at all times by experts in oral and maxillofacial radiology. The Sharpe's approach [26] was used to grade the severity of root resorption. Study models and data were encrypted to ensure that the investigator was not aware of the treatment categories during data collection and evaluation.CA has lately been used to treat a wide range of orthodontic issues [5]. However, there is still controversy in the literature regarding its accuracy in clinical praxis and compliance with the programmed actions [6]. Despite both devices' shortcomings, prior research has shown that both might be used to deal with cases of light and moderate overcrowding [7].

## Conclusion:

The decrease in thickness of alveolar bone was greater in orthodontic fixed appliances group as compared to clear aligners. The resorption of root at apical region was lesser in clear aligners group as compared to fixed appliances. The reduction in height of alveolar bone was observed in both therapies and it was almost similar in both categories.

## Figures and Tables

**Table 1:** Intra group variations in Bone thickness in fixed appliances group

	Labial				Palatal			
Fixed applainces	L1	L2	L3	Average	L1	L2	L3	Average
Pretreatment (Mean±SD)	0.88±0.38	1.11±0.30	1.08±0.75	1.08±0.61	1.66±0.79	3.26±1.08	4.84±1.70	3.34±1.85
Post treatment (Mean±SD)	0.71±0.59	1.10±0.69	1.28±1.11	1.10±0.85	1.22±0.85	2.50±1.25	4.10±2.17	2.87±1.98
P value	0.001	0.728	0.518	0.116	0.001	0	0.012	0

**Table 2:** Intra group variations in Bone thickness in clear aligners group

	Labial				Palatal			
L1	L2	L3	Average	L1	L2	L3	Average
Clear aligners
Pretreatment (Mean±SD)	0.85±0.47	0.90±0.48	1.11±0.42	0.95±0.46	1.52±0.63	3.01±1.25	4.75±1.97	3.16±1.93
Post treatment (Mean±SD)	0.71±0.66	0.96±0.55	0.92±0.74	0.86±0.66	1.22 ±0.81	3.06±1.24	4.82±1.69	3.04±1.99
P value	0.089	0.657	0.04	0.085	0.048	0.472	0.237	0.623

**Table 3:** Intra group variations in Bone height in fixed appliances group and clear aligners group

**Labial**	**Palatal**
Fixed applainces
Pre-treatment (Mean±SD) mm	2.03±0.60	1.52 ±0.77
Post treatment (Mean±SD) mm	2.97 ±1.61	2.41 ±1.89
P value	0	0
Clear aligners
Pre-treatment (Mean±SD) mm	2.11 ±0.66	2.21±0.66
Post treatment (Mean±SD) mm	3.21±2.46	3.11 ±2.46
P value	0	0

**Table 4:** Intra group variations in root resorption and inclination (MI -PA) in fixed appliances group and clear aligners group

	**Root resorption (mm)**	**Inclination (°)**
Fixed applainces		
Pre-treatment (Mean±SD)	11.83±1.79	119.35 ± 6.76
Post treatment (Mean±SD)	11.25±2.23	119.58 ± 8.49
P value	0	0.502
Clear aligners		
Pre-treatment (Mean±SD)	12.53±1.89	118.74 ±7.0
Post treatment (Mean±SD)	11.21±1.71	114.27 ± 5.53
P value	0.000**	0.044*

**Table 5:** Intergroup variations in bone thickness, bone height, root resorption and UI-PP

	**Bone thickness thickness (mm)**	**Bone height(mm)**	**Root resorption (mm)**	**MI-PA (°)**
Fixed appliances (Mean±SD)	0.21± 0.93	0.93±1.47	0.63±0.21	-1.24± 8.11
Clear aligners (Mean±SD)	-0.01±0.47	0.91±2.44	0.32±0.36	4.63±8.14
P value	0	0.16	0	0.03

**Table 6:** Prevalence and severity of apical resorption

	**Prevalence (%)**	**Severity of AR (%)**			
		0 degree	1 degree	2 degree	4 degree
Fixed applainces	82%	17.50%	80%	1.30%	1.30%
Clear aligners	68%	31.10%	67.50%	1.30%	0
The prevalence of resorption of root at apical region in OFA was 82% while it was 68% in OCA. More severe resorption of root at apical region was observed in OFA as compared to OCA (Table 6).
